# Vectors for expression of proteins with single or combinatorial fluorescent protein and tandem affinity purification tags in *Dictyostelium*

**DOI:** 10.1016/j.pep.2007.01.001

**Published:** 2007-06

**Authors:** Marcel E. Meima, Karin E. Weening, Pauline Schaap

**Affiliations:** School of Life Sciences, University of Dundee, MSI/WTB/JBC complex, Dow Street, Dundee DD15EH, UK

**Keywords:** Tandem affinity purification, Expression vector, Yellow fluorescent protein, *Dictyostelium discoideum*, Native protein complexes

## Abstract

We constructed a series of expression vectors for purification of native proteins and protein complexes in *Dictyostelium*. Protein purification is achieved by either a C-terminal or N-terminal fusion of the protein of choice to the tandem affinity purification (TAP) tag. The TAP tag consists of a protein A tag and a calmodulin binding peptide (CBP) and has been successfully used for purification of native protein complexes from yeast and animal cells. Protein expression is driven by the constitutive actin 15 promoter and the vectors optionally carry additional green- or yellow fluorescent protein (GFP or YFP) tags for fusion at either a C- or N-terminal location. Tandem affinity purification of native *Dictyostelium* protein complexes was tested by using pArc-34, one of the members of the well characterized *Dictyostelium* Arp2/3 complex, as bait. After denaturation and SDS–PAGE separation of the pArc-34 associated proteins all members of the Arp2/3 complex could be identified.

*Dictyostelium discoideum* is a genetically tractable model system for studying remodeling of the actin cytoskeleton during cell locomotion, cell division, phagocytosis and vesicle trafficking and for elucidation of signaling pathways that control chemotaxis and development. Several metazoan-type cytoskeletal components were identified for the first time in *Dictyostelium* [1–3], and the process of pleckstrin homology domain mediated recruitment that plays such crucial roles in leukocyte chemotaxis and phagocytosis was also first demonstrated in the Dictyostelids [4]. The *Dictyostelium* genome is now completely sequenced and each gene is therefore available for functional analysis by gene disruption, overexpression and mutagenesis [5]. Genetic approaches to create tagged mutations, such as REMI (restriction enzyme mediated integration), have identified numerous genes with crucial roles in developmental or cellular processes [6]. However in only a few cases has it been possible to use genetic approaches, such as screens for suppressor mutations, to determine epistatic relationships between genes [7]. Or, in other words, to determine in what order their cognate proteins act on each other in a pathway. In other genetically tractable systems, such as the fruit fly and the nematode, epistatic relationships are usually determined by crossing in loss- and gain-of-function mutants of genes in the pathway. The poor accessibility of the Dictyostelid sexual stage, the macrocyst, has prohibited this approach.

Other genetic screens, such as the yeast two-hybrid system or the split-ubiquitin method are being successfully used to identify interacting proteins in a pathway [8,9]. However, these methods depend on successful heterologous expression of the proteins in yeast, which is not possible for many proteins. The TAP tag was developed about seven years ago to purify native protein complexes from yeast [10,11] and was used for systematic identification of protein complexes in yeast [12–15] and metazoans [16,17]. For C-terminal tagging, the TAP tag contains in tandem, a calmodulin binding peptide tag, a TEV protease cleavage site and a protein A tag. For N-terminal tagging the order of these units is reversed. We have constructed a series of vectors for C- and N-terminal tagging of proteins expressed in *Dictyostelium*. Protein expression is driven by the strong constitutive actin15 promoter. Additional cloning sites were introduced to allow replacement of this promoter by an inducible promoter. A third green- or yellow fluorescent protein tag was introduced for C- or N-terminal expression in some of the vectors for rapid assessment of the levels of protein expression and the cellular location of the expressed proteins.

## Materials and methods

### Construction of plasmid vectors

Gene fragments were amplified by polymerase chain reaction (PCR)^2^ with a 4:1 mixture of the Taq and Pfu DNA polymerases (Promega, Madison, WI). All oligonucleotide primers used in this study are listed in Table 1. The integrating *Dictyostelium* vector EXP4(+) [18] was used as starting material. An *Xba*I and a *Hin*dIII site upstream of the actin15 promoter were first successively destroyed by filling in the overhanging sites after digestion with the Klenow fragment of polymerase I, followed by religation, resulting in vector EXP4(-XH). This also generated a unique *Nhe*1 site replacing the *Hin*dIII site.

A long multiple cloning site (MCS) was generated by deleting the existing polylinker by *Bgl*II/*Xho*I digestion of the vector and by ligating a duplex of primers MCS1 and MCS2 that carries compatible sticky ends into the *Bgl*II and *Xho*I sites. The resulting vector, EXP5(+) was used as the backbone for all subsequent constructs containing TAP and/or EGFP/EYFP fragments (Table 2).

The N-terminal TAP fragment (NTAP) was amplified from the plasmid pBS1761 [11] using primers NTAP1 and NTAP2 and ligated into the *Bam*HI and *Hin*dIII sites of EXP5(+), yielding pDV-NTAP. The C-terminal TAP fragment (CTAP) was amplified from plasmid pBS1479 [10] using primers CTAP1 and CTAP2 and ligated into the *Cla*I and *Xho*I sites of EXP5(+), yielding vector pDV-CTAP. Enhanced yellow- and green fluorescent protein genes (YFP) and (GFP) were amplified from the vectors pEYFP-N1 and pEGFP-C3 (Clontech, Mountain View, CA), respectively. For N-terminal location of YFP, primers tapYFP1 and tapYFP2 were used and insertion occurred in the *Hin*dIII and *Spe*I sites of EXP5(+), yielding pDV-NYFP. For C-terminal location of YFP or GFP, primers tapYFP3 and tapYFP4 were used and insertion occurred into the *Eco*RI and *Cla*I sites of EXP5(+), yielding pDV-CYFP or pDV-CGFP. All constructs were validated by sequencing across the entire tag and MCS and are listed in Table 2.

### Generation and transformation of construct p34-ArcCTAP

The *Dictyostelium* p34-Arc protein is encoded by the *ArpE* gene [5,19]. The 1251 bp *ArpE* coding region was amplified by PCR from genomic DNA isolated from wild-type AX2 cells, using primers p34Arc1 and p34Arc2 (Table 1). The *Bam*HI/*Xba*I digested PCR product was ligated into the *Bam*HI and *Xba*I sites of vector pDV-CTAP, yielding vector p34-ArcCTAP. The p34-ArcCTAP, pDV-CGFP-CTAP and pDV-CTAP constructs were transformed by electroporation into *D. discoideum* strain AX2 and transformants were selected by growth in the presence of 20 μg/ml G418.

### Purification of TAP-tagged protein complexes

TAP purification was performed as described previously [10,11] with some modifications. Briefly, 2 × 10^9^ exponentially growing AX2 cells expressing TAP constructs were harvested, washed twice in 10 mM Na/K-phosphate buffer, pH 6.5 and resuspended in 10 ml IPP (150 mM NaCl in 10 mM Tris, pH 8.0), containing one tablet of Complete Protease inhibitor cocktail (Roche, Lewes, UK) per 50 ml. Cells were lysed by addition of Triton X-100 to 1% (v/v) and incubated for 30 min on ice. Lysates were cleared by centrifugation for 20 min at 20000*g* and 4 °C. The supernatant was rotated for 1 h at 4 °C with 0.1% (v/v) Nonidet P-40 (NP-40) and 200 μl bed volume of IgG-agarose beads (Sigma, St Louis, MO) in Polyprep columns (Bio-rad, Hercules, CA). The beads were washed three times with IPPN (0.1% NP-40 in IPP), and once with 10 ml TEV cleavage buffer (1 mM DTT and 0.5 mM EDTA in IPPN). Bound complexes were liberated by rotating the column for 2 h at 16 °C with 100 units of TEV protease (Invitrogen, Carlsbad, CA) in 1 ml TEV cleavage buffer and recovered by elution.

Subsequently 3 μl of 1 M CaCl_2_, 3 ml CaM binding buffer (1 mM imidazole, 1 mM Mg-acetate, 10 mM β-mercaptoethanol and 2 mM CaCl_2_ in IPPN) and 200 μl of bed volume Calmodulin Affinity Resin (Stratagene, La Jolla, CA) were added per milliliter of eluate. The mixture was rotated for 1 h at 4 °C in a second Polyprep column. Beads were washed 3 times with 10 ml CaM binding buffer and bound material was eluted in 5 fractions of 200 μl each with CaM elution buffer (1 mM imidazole, 1 mM Mg-acetate, 10 mM β-mercaptoethanol and 2 mM EGTA in IPPN). Proteins were separated by SDS–PAGE on 4–12% Bis–Tris gels (Invitrogen, Carlsbad, CA) and visualized with a Colloidal Blue Staining kit (Invitrogen, Carlsbad, CA).

### Protein mass fingerprinting

Protein mass fingerprint data was obtained by MALDI-TOF-TOF (MS/MS) analysis performed at the University of Dundee ‘Fingerprints’ Proteomics Facility using an Applied Biosystems 4700 Proteomic Analyser. Protein bands were excised, in-gel reductively alkylated and digested in 20 mM NH_4_HCO_3_ containing 0.1% *N*-octylglucoside and 12.5 μg/ml trypsin [20]. One-tenth of each digest was applied to a 192 well MALDI sample plate (Applied Biosystems (AB)), allowed to air dry and mixed with 0.5 μl of a solution of 5 mg/ml α-cyano-4-hydroxy-*trans*-cinnamic acid matrix (Sigma, StLouis, USA), 50% (v/v) acetonitrile, 0.1% (v/v) trifluoroacetic acid in 10 mM NH_4_H_2_PO_4_, and then allowed to air dry prior to analysis.

The mass spectrometer was internally calibrated using the AB 4700 Proteomic Analyser Calibration Mix. Using the 4000 series Explorer Software (AB), MS spectral data were acquired from the samples and an MS/MS list was automatically generated for further analysis based on the top 5 most intense ions present (trypsin and major keratin ions were excluded). The MS and MS/MS spectral data obtained were exported from the 4700 using the Global Proteome Server (GPS) Explorer Software (AB). The data were then submitted to a local MASCOT search engine (Matrixscience, London, UK) for protein database searching against the Uniprot database (http://www.ebi.ac.uk/swissprot/) for identification.

## Results and discussion

### Cloning strategies

**Fig. 1:**
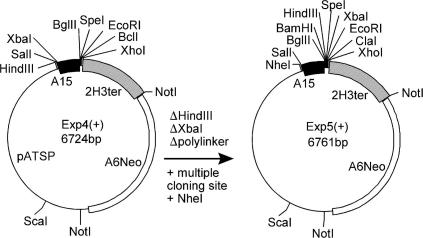
Construction of the primary vector Exp5(+). The *Xba*I and *Hin*dIII sites that precede the actin15 promoter in the existing *Dictyostelium* expression vector Exp4(+) were filled in and religated, which deleted both sites and created a novel unique *Nhe*I site. The small polylinker of Exp4(+) was subsequently exchanged with a larger multiple cloning site in order to accommodate dual TAP and YFP/GFP tags with at least three more restriction sites to insert the gene of choice.

The integrating *Dictyostelium* vector EXP4(+) was chosen as the backbone for all constructs. This vector is derived from pATSP [21], itself a derivative of the standard cloning vector pAT153. EXP4(+) carries the Act6NeoR cassette for G418 selection in *Dictyostelium*, and a cassette for protein expression consisting of the constitutive actin 15 (A15) promoter, the 2H3 terminator and a small polylinker with 6 restriction sites [18]. Because some of the cloning steps used to make this vector could not be retraced, we sequenced about 900 bp each around the points of fusion of the 2H3 terminator with the actin6 promoter, and the NeoR terminal region with the original pAT153 vector backbone. This revealed that the 2H3 terminator was preceded by 257 bp of the 2H3 coding region. However, since this coding sequence is downstream of the stopcodon in EXP4(+) and all derived vectors, it is not incorporated in the expressed tagged proteins. The complete sequence of EXP(4+) was reconstructed and a vector map is presented in Fig. 1.

To obtain a larger multiple cloning region, we first successively destroyed the *Xba*I and *Hin*dIII sites upstream of the A15 promoter, creating a novel *Nhe*I site. In addition to the existing *Sal*I site, this site can be used to exchange promoters or to insert genes with their own promoter. The existing polylinker was removed by *Bgl*II and *Xho*I digestion and replaced by an adaptor duplex of oligonucleotides MCS1 and MCS2 (Table 1), yielding vector EXP5(+) (Fig. 1). The new multiple cloning site (MCS) contains an ATG start codon, preceded by 5 adenine residues to generate the *Dictyostelium* Kozak sequence and followed by 7 unique restriction sites:

The MCS was subsequently used to clone in the TAP tags and enhanced YFP or GFP tags that were amplified by PCR. This is possible in any configuration i.e. TAP alone, YFP(GFP) alone or combinations of TAP and YFP(GFP) at the same site or at opposing sites. When cloned at the same site, the YFP(GFP) is in between the gene of interest and the TAP sequence as indicated above. Here, NTAP and CTAP stand for N- and C-terminal TAP, respectively, while NYFP and CYFP(GFP) stand for N- and C-terminal GFPs and YFPs. Vectors with single NTAP, CTAP, NYFP or CYFP(GFP) tags were created first and validated by DNA sequencing across the entire tag and MCS. The CTAP fragment contained a stopcodon before the *Xho*I restriction site. Vectors with TAP + GFP(YFP) tags were then generated by removing a tag from one of these vectors and cloning it in the appropriate position in the other vectors. Some restriction sites cannot be used anymore: *Bgl*II will digest both the NTAP and the CTAP tags, while *Eco*RI will digest the CTAP tag. *Nhe*I will digest the CTAP tag, which then leaves only *Sal*I to introduce a different promoter. All vectors prepared in the course of this work are listed in Table 2 and their sequences are deposited in GenBank.

### Purification of the Dictyostelium Arp2/3 complex with TAP-tagged p34-Arc

**Fig. 2:**
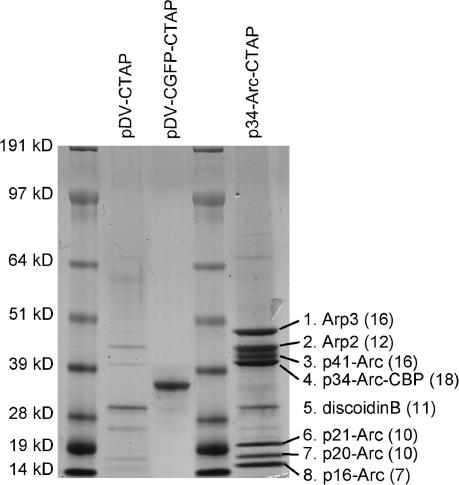
TAP mediated pull-down of the Arp2/3 complex. Extracts of *Dictyostelium* cells transformed with vector pDV-CTAP, pDV-CGFP-CTAP or p34-Arc-CTAP were subjected to the TAP purification protocol and size-fractionated by electrophoresis on SDS–PAGE. Gels were stained with colloidal blue and the eight protein bands that were purified from the p34-Arc-CTAP transformed cells were cut out and subjected to tryptic peptide fingerprinting by mass spectrometry. The number of different peptides obtained for each band is indicated in parentheses. Query of the Uniprot protein database with the obtained peptide sequences yielded all 7 members of the *D.discoideum* Arp2/3 complex and discoidin B.

To validate that the TAP tag can be used to purify native protein complexes in *Dictyostelium* we used a TAP tagged *D. discoideum* p34-Arc protein as bait for purification of members of the well-characterized Arp2/3 complex. The Arp2/3 complex is a complex of 7 proteins, including the p34-Arc protein, involved in the nucleation of actin polymerization [19]. We prepared a C-terminal fusion of the TAP tag with *D. discoideum* p34-Arc, as was previously done for yeast and human p34-Arc (named Arc35 and ARP2C, respectively) [14]. The p34-Arc gene was amplified from *D. discoideum* genomic DNA using the primers p34Arc1 and p34Arc2 (Table 1), which harbour *Bam*H1 and *Xba*I sites, respectively, and cloned into the *Bam*HI and *Xba*I digested pCTAP vector. This yielded the vector p34-Arc-CTAP, which was transformed into *D. discoideum* AX2 cells. AX2 cells transformed with the pDV-CTAP vector and with a pDV-CGFP-CTAP vector served as controls. For each transformed cell line, lysates were prepared by detergent treatment from 2 × 10^9^ cells and adsorbed to IgG agarose. After extensive washing, the 4.8 kD CBP moiety of the TAP tag with fused “bait” and complexed “prey” were released from IgG agarose by TEV protease treatment. The proteins or protein complexes were further purified over calmodulin resin, eluted with EGTA, denatured and size-fractionated on SDS–PAGE gels. Fig. 2 shows that a few faint bands were visible in preparations from cells that contained the empty pDV-CTAP vector. Extracts from pDV-CGFP-CTAP transformed cells showed a strong band at 32 kD, the expected size of the CGFP–CBP fusion (26.8 + 4.8 kD) after successful cleavage. Extracts of cells transformed with p34-Arc-CTAP displayed a pattern of 8 bands, similar to that observed after purification of the *Dictyostelium* Arp2/3 complex by traditional chromatography [19]. Bands 1–8 were cut out from the gel, proteins were digested in gel with trypsin and sequences of the extracted peptides were determined by MALDI-TOF/TOF mass spectrometry. This yielded 12–18 different peptide sequences for each of the 4 larger bands and 7–11 different peptide sequences for each of the four smaller bands. Query of the Uniprot protein database with the peptide sequences yielded all members of the *D. discoideum* Arp2/3 complex for bands 1–4 and 6–8 (Fig. 2). Band 5, which is also present in the pDV-CTAP and pDV-CGFP-CTAP extracts was identified as discoidin B, a highly abundant *Dictyostelium* lectin, which is most likely a non-specific contaminant.

## Conclusions

We have constructed eleven integrating plasmid vectors for protein expression in *Dictyostelium* that carry the TAP tag for tandem affinity purification of native proteins or protein complexes. In addition to the TAP tag for N-terminal or C-terminal fusion, a subset of vectors carry an additional enhanced GFP or enhanced YFP tag at either the same or opposite location as the TAP tag. The vectors are constructed in such a manner that the existing constitutive actin15 promoter can easily be swapped for an inducible promoter. Alternatively the pDV-CTAP vectors also allow cloning of a gene under its own promoter. In addition, the neomycin selection cassette can be exchanged for a different selection marker e.g. blasticidin resistance, which requires only a single copy of the *bsr* gene.

Preferentially, the “bait” gene of choice should be expressed under its own promoter from a single copy vector in a “bait” null mutant. This will avoid artefacts due to ectopic expression and competition of the endogenous “bait” with the tagged “bait” for binding to cellular proteins. Moreover, rescue of the null mutant phenotype by the tagged “bait” will ensure that the tags are not interfering with protein function. However, it may often be desirable to overexpress the “bait” to obtain a sufficient yield of protein complexes, although this also increases the probability of pulling down unrelated proteins.

We show that for the pDV-CGFP-CTAP vector, the highly fluorescent GFP protein is efficiently released and purified by the two affinity steps and the TEV protease treatment. We also show that by using a p34-Arc-CTAP fusion protein as bait, the 6 associated members of the Arp2/3 complex could be isolated with negligible contamination. We therefore expect that these vectors will prove to be useful tools for identification of native protein complexes in *Dictyostelium*.

## Figures and Tables

**Table 1:** Oligonucleotide primers used in this work

Name	DNA sequence
MCS1	5′-GATCTAAAAAATGGGATCCAAGCTTACTAGTTCTAGAGAATTC ATCGATTAAC-3′
MCS2	5′-TCGAGTTAATCGATGAATTCTCTAGAACTAGTAAGCTTGGATC CCATTTTTTA-3′
NTAP1	5′-GCAGGATCCGCAGGCCTTGCGCAACACG-3′
NTAP2	5′-CCGTAAGCTTATCGTCATCATCAAGTGCC-3′
CTAP1	5′-GCATCGATGAAAAGAGAAGATGGAAAAAGAATTTCATAGCCG-3′
CTAP2	5′-GCTCTCGAGTTAGGTTGACTTCCCCGCGGAATTCG-3′
tapYFP1	5′-GGTCAAGCTTGTGAGCAAGGGCGAGGAGCTG-3′
tapYFP2	5′-GCACTAGTCTTGTACAGCTCGTCCATGCCG-3′
tapYFP3	5′-GTCGAATTCGTGAGCAAGGGCGAGGAGCTG-3′
tapYFP4	5′-GCCATCGATCTTGTACAGCTCGTCCATGCCG-3′
p34Arc1	5′-GCGGGATCCTTATTATTAGAAACACACAATCG-3′
p34Arc2	5′-GTCTCTAGAATTTTGTTTAAAGAATTTACCAGTGATTG-3′

**Table 2:** Plasmid vectors that were used or prepared in this work.

Vector name	Backbone	Tag position	Accession #
EXP4(+)	pAT153	None	EF028663
EXP5(+)	EXP4(+)	None	EF028664
pDV-NYFP	EXP5(+)	N-terminal YFP	EF028665
pDV-NTAP	EXP5(+)	N-terminal TAP	EF028666
pDV-CGFP	EXP5(+)	C-terminal YFP	EF028667
pDV-CYFP	EXP5(+)	C-terminal GFP	EF028668
pDV-CTAP	EXP5(+)	C-terminal TAP	EF028669
pDV-NTAP-NYFP	EXP5(+)	N-term. YFP and TAP	EF028670
pDV-NYFP-CTAP	EXP5(+)	N-term. YFP, C-term. TAP	EF028671
pDV-CGFP-CTAP	EXP5(+)	C-term. GFP, C-term. TAP	EF028672
pDV-CYFP-CTAP	EXP5(+)	C-term. YFP, C-term. TAP	EF028673
pDV-NTAP-CGFP	EXP5(+)	N-term. TAP, C-term. GFP	EF028674
pDV-NTAP-CYFP	EXP5(+)	N-term. TAP, C-term. YFP	EF028675

## Footnotes

2 *Abbreviation used:* PCR, polymerase chain reaction; TAP, tandem affinity purification; CBP, calmodulin binding peptide; GFP, green fluorescent protein; YFP, yellow fluorescent protein; CTAP, C-terminal TAP fragment; NTAP, N-terminal TAP fragment; MCS, multiple cloning site.
